# Factors Underlying Individual Differences in Speech-Recognition Threshold (SRT) in Noise Among Older Adults

**DOI:** 10.3389/fnagi.2021.702739

**Published:** 2021-07-05

**Authors:** Larry E. Humes

**Affiliations:** Department of Speech, Language, and Hearing Sciences, Indiana University, Bloomington, IN, United States

**Keywords:** aging, hearing loss, speech perception, speech in noise (SIN), cognition

## Abstract

Many older adults have difficulty understanding speech in noisy backgrounds. In this study, we examined peripheral auditory, higher-level auditory, and cognitive factors that may contribute to such difficulties. A convenience sample of 137 volunteer older adults, 90 women, and 47 men, ranging in age from 47 to 94 years (*M* = 69.2 and SD = 10.1 years) completed a large battery of tests. Auditory tests included measures of pure-tone threshold, clinical and psychophysical, as well as two measures of gap-detection threshold and four measures of temporal-order identification. The latter included two monaural and two dichotic listening conditions. In addition, cognition was assessed using the complete Wechsler Adult Intelligence Scale-3rd Edition (WAIS-III). Two monaural measures of speech-recognition threshold (SRT) in noise, the QuickSIN, and the WIN, were obtained from each ear at relatively high presentation levels of 93 or 103 dB SPL to minimize audibility concerns. Group data, both aggregate and by age decade, were evaluated initially to allow comparison to data in the literature. Next, following the application of principal-components factor analysis for data reduction, individual differences in speech-recognition-in-noise performance were examined using multiple-linear-regression analyses. Excellent fits were obtained, accounting for 60–77% of the total variance, with most accounted for by the audibility of the speech and noise stimuli and the severity of hearing loss with the balance primarily associated with cognitive function.

## Introduction

The World Health Organization (WHO) estimated that there are 162 million older adults worldwide with “disabling” age-related hearing loss (Stevens et al., 2013). World Health Organization (WHO) (2021) estimates the prevalence of such disabling hearing loss to be 25% for those over 60 years of age, increasing from 15.4% globally among people aged in their 60s to 58.2% globally for those over 90 years of age. “Disabling” hearing loss was defined by the WHO in both reports as a pure-tone average at 500, 1,000, 2,000, and 4,000 Hz (PTA4) in the better ear ≥35 dB HL. According to the current WHO hearing-impairment grade system, the same one used by Stevens et al. (2013), the onset of “disabling” hearing loss corresponds to those having a moderate hearing impairment. If one were to include those with mild hearing impairments, defined on this same scale as better-ear PTA4 between 20- and 35-dB HL, then the prevalence of age-related hearing loss increases from 25% to 65%. There is mounting evidence that those with such mild impairments, and even those in the “normal hearing” category (PTA4 ≤ 20 dB HL), have significant communication difficulties and often benefit from intervention with hearing aids (Ferguson et al., 2017; Humes et al., 2017, 2019; Humes, 2020a, 2021a).

The loss of hearing sensitivity with aging, as captured *via* pure-tone audiometry, is well established. So much so that there is an ISO standard describing the progression of hearing loss throughout adulthood for both men and women (International Standards Organization, 2017). It has also been recognized for many years that age-related hearing loss has a significant negative impact on speech communication. Plomp (1978) provided a synthesis and analysis of much of this early literature regarding the impacts of age-related hearing loss on speech communication arguing that there were two distinct components to the speech-communication difficulties experienced by older adults, one captured by speech perception in quiet and the other by speech perception in noise. This two-component model of speech perception was described earlier by Carhart (1951) and Carhart and Tillman (1970), but the model by Plomp (1978) offered a much more complete and detailed characterization of these two components. Speech perception in quiet was driven almost entirely by the inaudibility of the speech signal arising from the measured pure-tone hearing loss and there has been broad consensus about this in the literature, both prior to Plomp (1978) and since (e.g., Humes and Dubno, 2010).

The factors underlying speech perception in noise, however, were modeled by Plomp (1978) to involve more than the inaudibility of the speech signal. In Plomp’s model, the perception of speech in noise was attributed to a distinct “distortion” factor whereas speech perception in quiet resulted primarily from a separate “attenuation” factor and, to a lesser extent, contributions from the same “distortion” factor. The ensuing decades witnessed a wide search for factors and mechanisms that might underlie the “distortion” factor, beginning with peripheral factors such as poor cochlear filtering (e.g., Festen and Plomp, 1983; Dreschler and Plomp, 1985), and progressing to higher-level cognitive processes (e.g., van Rooij et al., 1989; van Rooij and Plomp, 1990, 1992; George et al., 2007; Humes and Dubno, 2010). In general, many of the peripheral supra-threshold deficits observed in older listeners with impaired hearing proved to be more a function of elevated thresholds than the presence of cochlear pathology (e.g., Levitt, 1971; Ludvigsen, 1985; Reed et al., 2009; Desloge et al., 2010, 2012). Humes et al. (2012), reviewing the literature on the speech-communication problems of older adults over the preceding 20 years, concluded that the inaudibility of the speech signal is a key factor, but additional deficits in auditory temporal processing and cognitive function often make substantial contributions to the problems experienced by older adults, especially when listening to speech in a background of competing speech or speech-like noises.

Humes et al. (2012), making use of the taxonomy of hypotheses underlying the speech-understanding problems of older adults proposed initially by CHABA (1988) and re-emphasized by Humes (1996), noted that there was considerable support for two of the three hypothesized mechanisms: peripheral and cognitive. The third hypothesized mechanism, central-auditory, was more challenging to support with the evidence available. This was due, in large part, to the confounding of measures supporting “central auditory” factors by peripheral hearing loss, cognitive function, or both. For example, dichotic processing of syllables or words, often considered to be a “central auditory” measure, can be impacted both by peripheral hearing loss, making the recognition of the speech signals more difficult, and by cognitive processes, such as selectively attending to one ear or dividing attention between the two ears (e.g., Cherry, 1953; Bronkhorst, 2000, 2015; Humes et al., 2006). Likewise, some “central auditory” tasks involving the processing of brief and rapid stimuli, such as the perception of time-compressed speech, may again be impacted negatively by both peripheral hearing loss and cognitive speed of processing (Humes et al., 2007; George et al., 2007). As a result, Humes et al. (2012) suggested that such behavioral speech-perception measures might be better referred to as measures of "higher level" auditory processing leaving open the possibility that they may be cognitive in nature rather than modality-specific central-auditory measures.

The detailed review by Humes et al. (2012) highlighted evidence from several studies that supported the primary contributions of age-related hearing loss to the speech-understanding problems of older adults, especially for unaided listening. That report, however, also noted considerable evidence in support of age-related changes in auditory temporal processing and cognitive function and their significant additional contributions to the speech-understanding problems of older adults, especially in backgrounds of competing speech. A meta-analysis of 25 studies by Dryden et al. (2017), which included a wide range of cognitive measures, found that cognitive factors consistently explained about 9% of the variance in unaided speech-in-noise performance of adults (age > 18 years) most of whom had hearing thresholds in the normal-to-mild severity range.

As noted in reviews by Akeroyd (2008) and Humes and Dubno (2010), the importance of auditory temporal processing and cognition tended to increase once the audibility deficit had been overcome through amplification. Humes and Dubno (2010) noted that this was particularly true when competing speech or fluctuating speech-like noise was the competing sound. Other more recent studies with relatively large sample sizes ranging from about 100–200 older adults support these conclusions. Humes et al. (2013), for example, used spectral shaping to ensure full audibility of speech through at least 4000 Hz and found that individual differences in sentence identification and recognition were largely determined by several higher-level processing factors which combined accounted for 59% of the variance.

Similarly, Rönnberg et al. (2016) obtained several measures of higher-level auditory processing and multiple measures of speech perception in steady-state noise and multi-talker backgrounds with the speech and noise stimuli amplified by an experimental hearing aid. All 200 older adults were experienced hearing-aid users with long-standing mild-to-moderate hearing loss. Rönnberg et al. (2016) found that 10–40% of the variance in aided speech understanding could be explained by hearing loss, temporal fine structure, and cognition, with hearing loss tending to have the greatest weight even for aided listening.

Bernstein et al. (2016) in an analysis of data from 153 adults (mean age = 59.9 years) found that performance on a spectrotemporal modulation-detection task accounted for significant amounts of variance (28%) beyond that accounted for by hearing thresholds from 2,000–6,000 Hz (31%) for sentence recognition in multi-talker competition. Again, spectral shaping of the speech and competition was applied individually *via* a master hearing aid.

Most recently, Nuesse et al. (2018) examined the recognition of sentences in a variety of backgrounds, including cafeteria noise, for 41 adults ranging in age from 60 to 77 years. Participants were divided into two groups: elderly normal hearing (ENH), and elderly hearing impaired (EHI). For the ENH group, a single cognitive factor (of four included) was the lone significant predictor of speech recognition for two of the five speech-recognition conditions. In one case, the lone cognitive predictor was attention and in the other, it was the speed of lexical access. For the EHI group, on the other hand, hearing loss was the lone significant predictor for all five speech-recognition conditions and accounted for 38–55% of the variance. The authors note that this was true despite the use of hearing aids matched to NAL-NL2 gain prescription targets for each listener. It should be noted, however, that NAL-NL2, as is true of most gain-prescription procedures, does not optimize the audibility of speech, at least as quantified with the Speech Intelligibility Index (SII; American National Standards Institute, 1997; Humes and Dubno, 2010). The SII ranges from 0 to 1.0 and basically reflects the proportion of the speech signal that is optimally audible. For the Nuesse et al. (2018) study, the aided SII for the speech signal alone at 65 dB SPL was calculated here, based on the median audiograms in that study, to be 0.96 and 0.70 for the ENH and EHI groups, respectively. Thus, hearing loss substantially affected the audibility of the aided speech spectrum for the EHI group but not the ENH group which may explain the differences in the predictors identified from the regression analyses for these two groups in the study by Nuesse et al. (2018).

The recent studies reviewed above all made use of spectrally shaped speech, either in the laboratory or *via* hearing aids matched to targets, to overcome the loss of audibility. When hearing aids matched to targets were used, high-frequency pure-tone thresholds always emerged as a significant predictor of performance (Bernstein et al., 2016; Rönnberg et al., 2016; Nuesse et al., 2018). When the speech and noise stimuli were shaped to optimize audibility through at least 4,000 Hz, then the relative importance of pure-tone threshold for the prediction of aided speech understanding in competing speech remained significant but diminished greatly (Humes et al., 2013). The difference in the relative importance of pure-tone thresholds to aided speech-understanding performance in noise between these two approaches to overcoming the inaudibility of speech and noise stimuli is expected based on the residual inaudibility following amplification to clinical gain targets (Humes, 2007).

As discussed in detail in Humes (2007), however, there are at least two interpretations of the correlations of pure-tone thresholds with speech-in-noise performance. The most obvious is that the sloping high-frequency hearing loss common in aging renders the low-intensity, high-frequency components of speech inaudible, much like low-pass filtering. The sensorineural hearing loss associated with aging, however, is not a simple attenuation as might occur if the loss were conductive in nature (as in low-pass filtering). Rather, the underlying cochlear pathology that causes the elevation in thresholds in older adults may also produce other peripheral processing deficits and the severity of the pure-tone hearing loss may serve as a marker for the corresponding severity of that underlying cochlear pathology. Humes (2007) suggested several ways in which these two impacts of hearing loss might be disentangled, including the use of amplified speech to overcome the inaudibility associated with the elevated pure-tone thresholds.

Of the various approaches to overcoming the inaudibility of the speech and noise stimuli, increasing the overall level of the stimuli is perhaps the simplest and has been used most frequently over the years (Humes, 2007). Often, however, depending on the stimulus level used, it does not fully restore the audibility of the higher frequency regions of the speech and noise stimuli in older adults with age-related hearing loss. As was noted above, residual inaudibility also often occurs in studies using master hearing aids and clinical prescription targets. The SII, however, offers a way to quantify the residual inaudibility of the speech and noise stimuli. Although the SII can be expected to be correlated with PTA4, there is not a one-to-one association between the two measures. If high speech levels are used, for example, there will be no impact of hearing loss on speech audibility until a specific amount of hearing loss has been reached at a given frequency. Specifically, until the hearing loss reaches a level that is 15 dB below the RMS long-term-average speech spectrum, the hearing loss has no impact on speech audibility and the SII is unaffected (Humes and Dubno, 2010). As hearing loss at a given frequency exceeds this level, every decibel of increase in hearing loss reduces the contributions of that frequency region to the SII until the hearing loss exceeds a level corresponding to 15 dB above the RMS long-term-average speech spectrum. Thus, there will be a strong correlation between the measured hearing loss and the SII only for thresholds that fall in the 30-dB band within ± 15 dB of the RMS long-term-average speech spectrum.

The correspondence between PTA4 and the SII is further complicated due to the differential weighting of frequency regions by both metrics. The PTA4 calculation, for example, weights the hearing loss at 500, 1,000, 2,000, and 4,000 Hz equally through use of the simple arithmetic average of the hearing thresholds at these four frequencies. The SII, however, weights each frequency differently, generally ascribing the highest weights to the region of 2,000–4,000 Hz, although this varies with the nature of the speech materials (American National Standards Institute, 1997). Moreover, the presence of background noise can have an impact on the specific hearing threshold at a given frequency that impacts speech audibility. Finally, the SII captures the well-known negative effects of high presentation levels on speech that impact the performance even of young adults with normal hearing (American National Standards Institute, 1997). In summary, the correlations between the SRT and hearing loss observed in studies of the SRTs in noise among older adults, even when master hearing aids with clinical gain targets have been used, may incorrectly interpret the impact of those thresholds on speech audibility. That is, the use of “amplification” does not ensure that the full audibility of speech (and noise) has been restored for study participants. A more suitable metric of speech audibility in such studies is the SII.

In the present study, we obtained the speech-recognition threshold (SRT) in noise from 137 older adults with varying degrees of hearing loss. The SRT represents the speech level required for 50%-correct recognition of the speech stimulus. The speech materials were either sentences (QuickSIN; Killion et al., 2004) or monosyllabic words (WIN; Wilson, 2003). Each of these popular clinical SRT measures makes use of a female talker and multi-talker competition. They primarily differ in the amount of context provided, although the QuickSIN sentences are not considered to have rich semantic context (e.g., “It is a band of steel three inches wide”). These materials are typically presented at an overall level of 83 dB SPL (70 dB HL) in the clinic. Given the concerns about the audibility of the speech spectrum noted above, the nominal presentation level used here was 93 dB SPL. Consistent with WIN test administration guidelines, if the PTA for 1,000, 2,000, and 4,000 Hz was 40 dB HL or higher, the presentation level was raised 10 dB to 103 dB SPL. The SII was calculated for each participant to examine the contributions of speech audibility to the measured SRTs in noise.

Measures of higher-level auditory processing were also obtained from every participant. These included measures of temporal gap-detection threshold at two different frequencies and several measures of temporal-order identification for short vowel sequences, both monaural and dichotic. These psychophysical measures had been obtained from 245 young, middle-aged, and older adults previously (Humes et al., 2010; Busey et al., 2010) and more recently in a longitudinal follow-up of the original cross-sectional study (Humes, 2021b). In addition, two visual measures, one of temporal processing (flicker fusion) and one of text recognition akin to the auditory SRT, were also obtained here. Finally, full cognitive assessments using the Wechsler Adult Intelligence Scale-Third Edition (WAIS-III; Wechsler, 1997) were completed by all participants. After examining age-group differences by decade, linear-regression analyses were applied to examine the factors accounting for the individual differences in performance on each measure of SRT in noise.

## Materials and Methods

### Participants

A total of 137 adults (90 women, 47 men) with a mean age of 69.2 years (range of 47–94 years) participated in this study. Of the 137, 101 had completed the same cognitive and psychophysical measures included in this study 9 years earlier as part of a longitudinal study of sensory and cognitive changes (Humes, 2020b, 2021b). As noted in Humes (2021b), there were no learning or practice effects for the measures considered here that resulted from this prior testing. The measures of SRT in noise, the dependent measures of interest in this study, had not been included in the evaluation 9 years earlier.

At the time of *initial* entry into the study, currently, for 36 participants and 9 years prior for 101 individuals, participants were recruited *via* advertisements in the local newspaper, in bulletins or flyers for local community centers or organizations, or through existing laboratory databases of research volunteers. At initial entry into the study, the only selection criteria were based on age (40–89 years), a score ≥ 25 on the Mini Mental State Examination (MMSE; Folstein et al., 1975), and passing screens of sensory acuity. Maximum acceptable hearing thresholds and allowable visual acuity were established. Specifically, participants had to have corrected visual acuity of at least 20/40 based on an evaluation with a Snellen chart, hearing thresholds for air-conducted pure tones that did not exceed a maximum permissible value at each of several frequencies in at least one ear, and no evidence of middle-ear pathology in the test ear (air-bone gaps less than 10 dB and normal tympanograms). The maximum acceptable hearing thresholds (measured clinically) were: (1) 40 dB HL (American National Standards Institute, 2004) at 250, 500, and 1,000 Hz; (2) 50 dB HL at 2,000 Hz; (3) 65 dB HL at 4,000 Hz; and (4) 80 dB HL at 6,000 and 8,000 Hz. These limits were designed to make it likely that the psychophysical stimuli would be visible and audible when presented on subsequent tasks, but this was confirmed directly *via* identification screening. All participants were required to pass an identification screening of the four brief vowel stimuli in isolation, used in subsequent temporal-order measures, with at least 90% accuracy on one of up to four, 20-trial blocks. This was to ensure that listeners would be able to complete the subsequent temporal-order identification tasks which were targeting identification performance of either 50 or 75 percent correct (see below). If participants did not reach this 90% identification-accuracy criterion during screening, they were re-screened on a separate day. Participants ultimately unable to reach this criterion were not included in this study.

The 101 older adults who returned for the present study were not subjected to additional inclusion screening at the 9-year follow-up. The only requirement was that they were able to come into the laboratory for testing, could follow the task instructions, and could complete the required tasks. Although their hearing loss had progressed, as expected, over the intervening 9 years (Humes, 2021b), all but four could still identify the brief vowels used in the temporal-order identification tasks with at least 90% accuracy. For the four who were included in these analyses but scored below this initial study-entry criterion, the vowels were identified with 85%, 65%, 65%, and 60% accuracy in isolation.

Informed consent was obtained from all participants and they were paid $12–$15/h for their participation. This study was approved by the Indiana University Bloomington Institutional Review Board.

During the initial 2-h screening session, audiological examinations were completed, including pure-tone audiometry and immittance measures. Identification of the brief vowels used in the temporal-order sequence tasks was completed with each vowel presented individually and in quiet. A visual screen of distance vision was completed using a Snellen chart. A case history and the MMSE were also completed in this initial session. Finally, the two dependent measures in this study, the Words-In-Noise (WIN) test (Wilson et al., 2007) and the Quick Speech-In-Noise (QuickSIN; Killion et al., 2004) test were completed during this initial session. Eligible participants were then recruited for the main study and those volunteering to participate signed a second consent form for participation in the main study. The main study, involving auditory and visual psychophysical measures as well as a full cognitive evaluation, required an additional eight sessions with each session 2 h in duration.

### SRT in Noise: Materials and Procedures

Both the WIN test and the QuickSIN test were administered using the test CD and accompanying instruction manual. Both tests were administered using a clinical audiometer calibrated using the calibration track (Track 1) on each CD. Monaural SRTs in noise were obtained from each ear but only the SRTs from the right ear, the test ear for all monaural psychoacoustic measures, are discussed here. For the WIN and the QuickSIN, List 1 was used to obtain the SRT in noise. The QuickSIN was administered at 80 dB HL or 93 dB SPL. The QuickSIN list consisted of six sentences, each with five target words. The level of the sentences was fixed at an overall level of 93 dB SPL and the level of the multi-talker babble was varied in 5-dB increments from 25 to 0 dB SNR. The WIN was administered at the same level of 93 dB SPL for those with a pure-tone average at 500, 1,000, and 2,000 Hz (PTA) ≤ 40 dB HL or at 103 dB SPL for those with higher PTAs. For all conditions, the level of the multi-talker babble was fixed at 93 or 103 dB SPL, and the level of the speech varied to produce SNRs from 24 to 0 dB in 4-dB decrements, with five words presented at each SNR. It should be noted that both tests were administered at levels 10–20 dB higher than what is typically recommended clinically to minimize the impact of speech inaudibility and this was also the rationale for the use of two different levels for the WIN (Wilson, 2011). There were no complaints from the participants regarding the level of the stimuli being too loud.

### Auditory Psychophysical Measures: Materials and Procedures

For each of the psychophysical measures, a “threshold estimate” of performance was preceded by 20–40 familiarization trials, which included trial-to-trial feedback, and was obtained from three separate and stable blocks of trials that, when pooled, totaled 200–250 trials. The details of the stimuli and the psychophysical procedures for the auditory stimuli and procedures used here can be found in a series of prior studies (Humes et al., 2009; Humes and Dubno, 2010; Fogerty et al., 2010; Henshaw and Ferguson, 2013).

Next, the measures of auditory threshold sensitivity and gap detection were completed using an interleaved adaptive forced-choice psychophysical paradigm targeting 75% correct. For auditory threshold measurement, measures were obtained first for pure tones at 500 Hz, then at 1,400 Hz, and finally at 4,000 Hz. Similarly, measurement of gap-detection threshold began at the 1,000-Hz center frequency and then proceeded to the 3,500-Hz center frequency. Noise bands with 1,000-Hz bandwidth were used to obtain the gap-detection thresholds. This use of a fixed order reinforced the need for familiarization trials prior to each measure and for stable threshold estimates based on 200–250 trials.

Four temporal-order identification measures were then completed, each making use of the same set of four brief 70-ms vowel stimuli. Three of the four tasks required the identification of two-item sequences (e.g., “ah” “eh”) and one required the identification of a four-item sequence. The three two-item sequences differed regarding how the stimuli were presented to the subject with vowels in the sequence presented either to the same ear (monaural) or to different ears (dichotic). This manipulation was designed to explore lower-level (peripheral) vs. higher-level (central) auditory temporal-processing mechanisms. For example, for the auditory two-item dichotic task, the two sensory inputs cannot interact until the first auditory center in the brainstem processes inputs from both ears (the superior olivary complex). On the other hand, the same-ear monaural version of this task makes it possible for the interaction of the two stimuli in the sequence at a much lower-level, as low as the cochlea. For the two dichotic, two-item tasks, the difference between them was in the response required of the subject. In one case, the subject was required to identify the vowel sequence, just as in the monaural version of this task, whereas in the other case, the task was simply to identify which ear (right or left) was stimulated first. The latter temporal-order identification task was included because this is most often considered “temporal-order judgment” in the long history of interest in this measure (e.g., James, 1890; Fraisse, 1984) and, recently, the effects of aging on this form of temporal-order judgment have been examined (e.g., Babkoff and Fostick, 2017; Ronen et al., 2018). Finally, the monaural four-item sequence was included to increase the cognitive demands for this temporal-order identification task, thereby increasing the likelihood of uncovering an underlying cognitive factor. For all these auditory temporal-order measures, the threshold estimate obtained was the stimulus onset asynchrony (SOA) that was approximately midway between chance and 100% correct performance on the psychometric function relating performance to SOA. SOA is the time lag between the onsets of successive vowels in the sequence. Further details regarding the stimuli and procedures can be found elsewhere (Fogerty et al., 2010; Humes et al., 2010).

All auditory psychophysical testing was completed in a sound-attenuating booth meeting the ANSI S3.1 standard for “ears covered” threshold measurements (American National Standards Institute, 2003). Two adjacent subject stations were housed within the booth. Each participant was seated comfortably in front of a touch-screen display (Elo Model 1915L). The right ear was the test ear for all monaural measurements in this study. Stimuli were generated offline and presented to each listener using custom MATLAB software. Stimuli were presented from the Tucker-Davis Technologies (TDT) digital array processor with 16-bit resolution at a sampling frequency of 48,828 Hz. The output of the D/A converter was routed to a TDT programmable attenuator (PA-5), TDT headphone buffer (HB-7), and then to an Etymotic Research 3A insert earphone. Each insert earphone was calibrated acoustically in an HA-1 2-cm^3^ coupler (Frank and Richards, 1991). Output levels were checked electrically just prior to the insert earphones at the beginning of each data-collection session and were verified acoustically using a Larson Davis model 2800 sound level meter with linear weighting in the coupler monthly throughout the study. Prior to actual data collection in each experiment, all listeners received 10–30 practice trials to become familiar with the task. These trials could be repeated a second time to ensure comprehension of the tasks if desired by the listener, but this was seldom requested. All responses were made on the touch screen and were self-paced. Correct/incorrect feedback was presented after each response during experimental testing. Further methodological details, specific to each measure, can be found in prior studies (Humes et al., 2009; Humes and Dubno, 2010; Fogerty et al., 2010).

Several procedural steps were followed to minimize the impact of high-frequency hearing loss on auditory measures. Gap-detection thresholds, for example, were obtained using an overall presentation level of 91 dB SPL. For the four auditory temporal-order identification tasks, productions of four vowels that had the shortest duration, F2 < 1,800 Hz, and good identification during piloting were selected for stimuli. Stimuli were digitally edited to remove voiceless sounds, leaving only the voiced pitch pulses and modified in MATLAB using STRAIGHT (Kawahara et al., 1999) to be 70-ms long with a fundamental frequency of 100 Hz. Stimuli were low-pass filtered at 1800 Hz and normalized to the same RMS level. A single stimulus presentation measured 83 (±2) dB SPL and a presentation of two overlapping stimuli measured 86 (±2) dB SPL. All listeners completed the four temporal-order tasks in the following order: monaural two-item identification (Mono2), monaural four-item identification (Mono4), dichotic two-item vowel identification (DichID), and dichotic two-item ear or location identification (DichEar). For the three vowel-identification tasks, listeners were required to identify, using a closed-set button response, the correct vowel sequence exactly (i.e., each vowel in the order presented) for the response to be judged correct. The ear-identification task, DichEar, only required the listener to identify which ear (“Right” or “Left”) was stimulated first. The dependent variable measured was the stimulus onset asynchrony (SOA) between the presented vowels. Each threshold estimate for each temporal-order task was based on three valid estimates that were averaged together for analysis, resulting in a total of 216 (Mono2), 288 (Mono4), or 432 (DichID, DichEar) trials per SOA threshold estimate.

### Visual Psychophysical Measures: Procedures and Equipment

#### Flicker Fusion

Flicker fusion is a commonly used measure of visual temporal sensitivity threshold. Flicker sensitivity was determined by flickering a light around a constant mean luminance. Flicker frequencies of 2, 4, and 8 Hz were used. The depth of modulation around the mean luminance was adaptively varied to achieve a threshold contrast value in a modified two temporal interval task.

A custom-designed light box, in which six 60-watt incandescent bulbs back-projected onto a white translucent Plexiglas panel to produce an adapting surround of 112 candelas per meter squared (cd/m^2^). This panel was 57 cm × 57 cm, and in the center (behind the white Plexiglas panel) was a red light-emitting diode (LED) display device consisting of 12 LEDs that projected through three additional diffusing screens. The luminance was adjusted so that the mean luminance was 127.5 cd/m^2^. The display device cast a shadow of 10.78 degrees of visual angle and inside was the red circle of diameter 5.39°. Participants freely viewed the display at 53 cm with both eyes in a fully illuminated room (fluorescent lighting).

The stimuli were driven through a custom circuit and programmed *via* a 12-bit digital/analog (D/A) card (National Instruments PCI-6071e). Stimulus sequences were generated in MATLAB (Mathworks, MA) and sent to the D/A card *via* the Real Time Toolbox (Humusoft, Czech Republic). No auditory cues were perceptible from the operation of the device. The update rate was 1,000 Hz.

Participants were comfortably seated in front of the display. The experimenter initiated each trial. Only two intervals were used, marked by auditory recordings (“Test One” and “Test Two”). The experimenter initiated each trial, and the LEDs were modulated around the baseline 127.5 cd/m^2^ level at one of three frequencies (2, 4, or 8 Hz), which was embedded in a Gaussian temporal envelope 500 ms in duration. The effective visible duration was approximately 250 ms. The depth of modulation was varied according to two interleaved tracking programs with an initial step size for the first two reversals of each track of 0.25 and a final step size for the remaining seven reversals of each track set to 0.125. Contrast was defined as contrast = (luminance-127.5)/127.5. Note that this flicker task is not an absolute threshold task because the background luminance was set to 127.5 cd/m^2^ and the room lights were left on. The visual task should be viewed as a relative flicker judgment (i.e., which interval contained a steady light that appeared to “flicker”).

#### Text Recognition Threshold (TRT)

The TRT is a test of the ability to recognize written sentences that are partially obscured by a vertical grating. The Dutch version of the test, developed by Zekveld et al. (2007), was obtained and modified to present English sentences from the revised Speech in Noise (R-SPIN) test (Bilger et al., 1984). No other properties of the test were changed. On each trial, a row of equally spaced vertical black bars appeared then a sequence of words, that form a meaningful sentence, appeared behind (obscured by) the bars. The words appeared sequentially (250 ms per word) and the complete sentence remained on the screen for 3.5 s. The subject’s task was to read aloud as much of the sentence as he or she could identify. The difficulty of the task was varied adaptively (based on a subject’s performance) by increasing or decreasing the width of the bars (i.e., the percentage of unobscured text). The test consisted of four adaptive runs of 13 trials, with four different sets of R-SPIN predictability-high (PH) sentences. The threshold for each run was computed as the mean percentage of unobscured text on trials 5–13 and the final TRT value was the mean of the four threshold estimates.

### Cognitive Measures

#### A Quick Test (AQT)

The AQT was used to provide a measure of cognitive abilities that often decline with age (or due to various types of dementia) (Wiig et al., 2002). The test is designed to measure verbal processing speed (PS), automaticity of naming, working memory (WM), and the ability to shift attention between dimensions of multidimensional visual stimuli. The test consisted of three timed subtests in which subjects named the color and/or the shape of symbols arranged on a page in eight rows of five. Test 1 required subjects to name the color (black, red, blue, or yellow) of colored squares. The second test required subjects to name each shape on a page of black circles, squares, triangles, and lines. The third test included colored shapes (the same shapes and colors used in tests 1 and 2) and subjects were asked to name both the color and the shape. Subjects were told to proceed as fast and as accurately as they could and the total time to complete each subtest was recorded.

#### WAIS-III

The full 13-subscale version of the WAIS-III (Wechsler, 1997) was administered by a research assistant trained in test administration. Because this test makes use of auditory instructions or stimuli for various subscales, a personal “pocket talker” amplification system was available for use by the participant. It was used whenever the pure-tone average at 1,000, 2,000, and 4,000 Hz exceeded 25 dB HL or the participant complained of difficulty hearing the test administrator. All WAIS-III scale scores reported here are the raw scores rather than the age-corrected normed scores.

### SII Calculations

The SII was calculated for the WIN and the QuickSIN according to the methods described in ANSI S3.5 (1997). All calculations were made using the one-third octave-band method. When the long-term-average speech spectra of the WIN (Wilson et al., 2007) and the QuickSIN (Killion et al., 2004) were compared they were determined to be essentially equivalent and the QuickSIN speech spectrum was used in all calculations. For both tests, a female is the target talker and the competition is a multi-talker babble that has been shaped to match the spectrum of the speech. Therefore, the long-term-average noise spectrum was set to be the same as the QuickSIN speech spectrum as well. Given the high presentation levels used, all calculations included the level desensitization factor in the ANSI standard. In addition, for all SII calculations, pure-tone thresholds at octave intervals from 250 to 8,000 Hz from the audiogram were used to represent one-third-octave band thresholds (Cox and McDaniel, 1986) at those same octave center frequencies. Thresholds at the 250-Hz frequency were extrapolated to the bands at 160 and 200 Hz whereas thresholds for all other one-third-octave center frequencies were interpolated from the adjacent octave center frequencies. The lowest one-third-octave band used for the SII calculations performed here was 160 Hz and the highest was 8,000 Hz.

**Figure 1:**
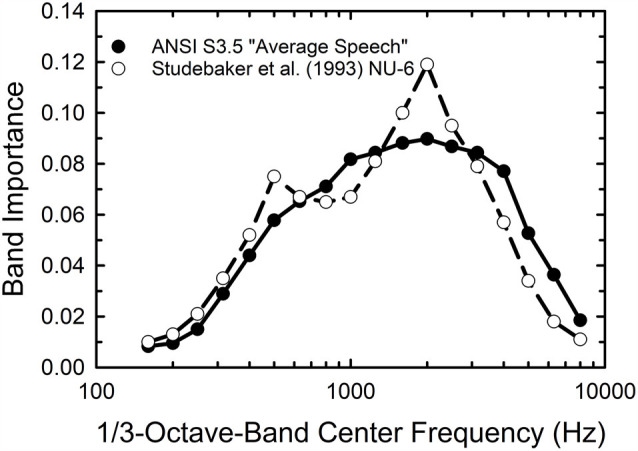
One-third octave-band importance weights for average speech (filled circles) and the Northwestern University Auditory Test No. 6 (NU-6) monosyllables from the American National Standards Institute (1997) standard for the Speech Intelligibility Index (SII).

A key component of the SII calculations is the band-importance function which ascribes differential weighting to the contributions of various one-third-octave bands of the speech spectrum. Based on the importance functions available in the ANSI standard it is likely that the weighting functions differ for the monosyllabic WIN and the sentence-based QuickSIN but such functions have not been derived for either test. Given that the WIN makes use of the NU-6 monosyllables the importance function derived for these materials by Studebaker et al. (1993) and included in the ANSI standard was used. Although the written materials are the same as in the WIN, the WIN involves a different recording and only a subset of all available NU-6 words. Nonetheless, it was considered the best choice available for the importance function for the WIN. For the QuickSIN, it was decided to use the importance function for “average speech” included in the ANSI standard. These two importance functions are compared in Figure 1. The NU-6 weights give somewhat greater importance to the frequency region 1,500–3,000 Hz than the function for average speech but, overall, they are more similar than dissimilar.

**Figure 2:**
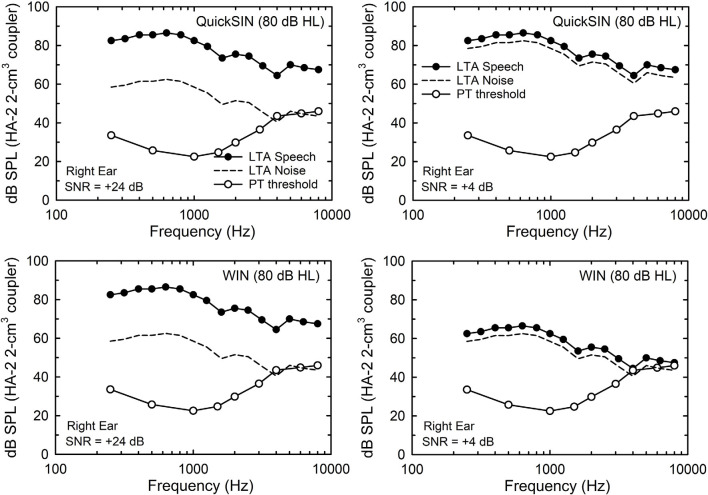
The 1/3-octave-band levels for the long-term average (LTA) speech (filled circles) and competing noise (multi-talker babble; dashed lines) for the QuickSIN (top) and WIN (bottom) for the 80-dB HL (93-dB SPL) presentation condition. In the left two panels, the signal-to-noise ratio (SNR) is +24 dB (the maximum for the WIN and within 1 dB of the maximum for the QuickSIN) and in the right two panels, the SNR is +4 dB (near-threshold level for young normal-hearing adults). Also shown in each panel are the mean pure-tone thresholds for the 137 older adults in this study (unfilled circles).

A key difference between the SRTs in noise obtained by the WIN and QuickSIN has to do with the way in which the speech and noise signals are presented and calibrated. For the WIN, the noise level is fixed at a level that is 24 dB below the maximum speech level to be used (+24 dB SNR). For the 93 dB SPL test level, at the maximum SNR of +24 dB, the noise is at 69 dB SPL. For the QuickSIN, it is the speech level that is fixed at the maximum level to be used and the noise level is reduced to lower levels to generate the desired SNRs. For the maximum SNR in the QuickSIN, +25 dB, the speech level is at the maximum, 93 dB SPL, in this study, and the noise is 25 dB lower at 68 dB SPL. The long-term-average speech and noise spectra for each test at an SNR of +24 dB for both are shown in the left two panels of Figure 2, along with the mean audiogram for the right ear from the 137 participants. Clearly, the underlying acoustics and audibility for both the QuickSIN (top) and WIN (bottom) are equivalent *at this high SNR*. However, the situation changes considerably at lower SNRs, such as the +4 dB SNR illustrated in the right-hand panels of Figure 2. Given the high and fixed speech level for the QuickSIN (top right), the audibility of the speech and noise spectra are not impacted by the hearing loss shown. For the WIN, however, at this same SNR of +4 dB, high-frequency hearing thresholds restrict the audibility of the speech and noise, although just slightly for this mean audiogram. Clearly, the SNR at SRT from the WIN is more likely to be impacted by the hearing loss than the QuickSIN and this reinforces the use of a 10-dB higher presentation level for the WIN for those with greater amounts of high-frequency hearing loss.

## Results and Discussion

### Effects of Age Group

Prior to examining the factors underlying individual differences in SRT in noise, the mean data were examined to establish the representativeness of the data sample in this study. Although this could be done by just pooling the data from all 137 older adults and comparing means to pooled means from prior studies of similarly aged participants, this was accomplished here by separating the 137 older adults into age decades from 50 to 59 through 80–89 years. In many prior studies, the sample sizes and age distributions were insufficient to segregate the data by age decade. Here, it was desired to both establish the representativeness of the current data by comparison of the aggregate data to those from prior studies while simultaneously presenting additional insights into the normative performance on each of these measures by age decade. Of course, sufficient numbers of participants are needed in each age decade to do so. Table 1 summarizes the composition of each of these subgroups. It should be noted that for the 50s age decade one individual was younger than the lower limit (47 years) and for the 80s decade, two individuals exceeded the upper age limit (ages of 90 and 94 years). With the sample sizes for each of the four age decades ranging from 26 to 41, there was sufficient data to assess performance by age decade while also evaluating the representativeness of the aggregate data.

**Table 1:** Age and gender details for each of the four age-decade groups formed.

Age Decade	*N*	Age M	Age SD	Age Min	Age Max	% Women
50s	31	56.2	3.0	47	59	74.2
60s	41	64.7	2.5	60	69	68.3
70s	39	74.9	2.9	70	79	59.0
80s	26	83.4	3.5	80	94	61.5
Total	137	69.2	10.1	47	94	65.7

*All age variables are in years. Note that for the age decade labeled “50s”, one individual was below the range of 50–59 years (age = 47 years) and for the decade labeled “80s”, two individuals exceeded the 80–89-year age range (ages = 90, 94 years)*.

**Figure 3:**
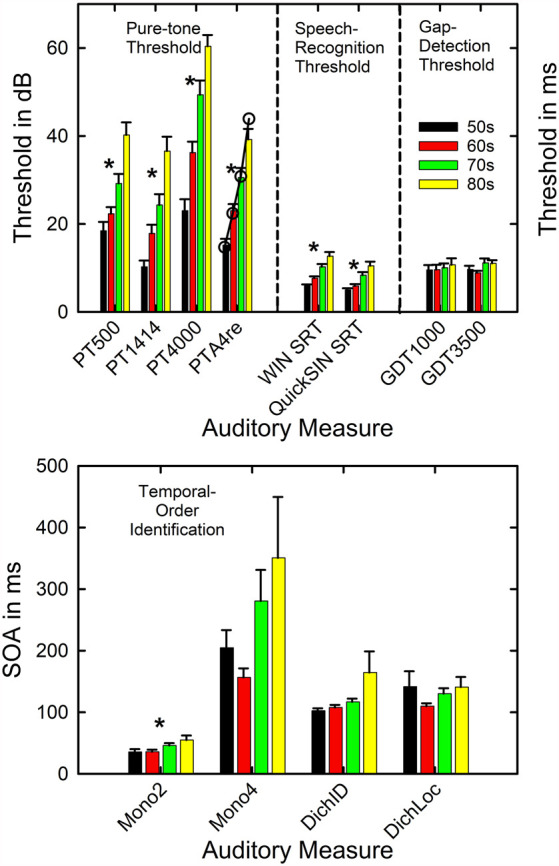
Means and standard errors are shown by age decade for each of the four types of auditory measures completed in this study. The asterisks mark those measures for which a significant effect of age decade was observed. The open circles in the top panel for PTA4re show comparison data from a population study of older adults in the US from Cruickshanks et al. (1998). PT = pure-tone threshold in dB SPL and 500, 1,414, and 4,000 represent the three test frequencies. PTA4re = pure-tone average for 500, 1,000, 2,000, and 4,000 Hz in the right ear in dB HL. SRT = speech-recognition threshold in dB; GDT = gap-detection threshold in ms. The x-axis labels in the lower panel show the four conditions for the temporal-order identification measures with the stimulus onset asynchrony (SOA) plotted in ms. Mono2 = monaural two-item. Mono4 = monaural 4-item. DichID = dichotic with vowel identification. DichLOC = dichotic with ear or location identification.

Figure 3 presents the means and standard errors for each of the four age decades for all the auditory measures obtained from the 137 adults in this study. General Linear Model (GLM) analyses were performed to examine the effects of age group on each of the measures in Figure 3. Asterisks mark significant (*p* < 0.05) differences among the age groups following Bonferroni adjustment for multiple dependent measures within a given type of auditory measure (e.g., *p* < 0.05/4 or 0.0125 for pure-tone threshold and temporal-order identification). As expected, hearing loss worsened significantly with age group, as shown in the top left panel, for both the laboratory psychophysical measures and the PTA4 for the right ear from the clinical audiogram (all *F*_(3,131)_ > 18.1, *p* < 0.001). The Eta-squared (η^2^) effect sizes for all four measures of hearing threshold, moreover, were very large (0.29 < η^2^ < 0.40; Cohen, 1988). *Post hoc* Bonferroni-adjusted *t*-tests revealed the following significant differences across age decades: (1) at 500 Hz and 1,400 Hz, mean thresholds for the 50-, 60- and 70-year-olds were better than those of the 80-year-olds and the thresholds for the 50-year-olds were better than those for the 70-year-olds; (2) at 4,000 Hz, the thresholds for each age decade differed from all others except for the 70- and 80-year-olds; (3) for the right-ear PTA4, all age decades differed significantly from one another. The unfilled circles superimposed on the bar graph for the right-ear PTA4 represent the mean values of the right-ear PTA4 for each age decade established for 1,244 older adults in the population sample of Cruickshanks et al. (1998). At least for this measure of hearing loss, the present data appear to be representative of older adults ranging in age from 50 to 89 years. Such population data or similarly large data sets do not exist for the other auditory measures included in Figure 3.

Given the significant differences in hearing thresholds across the age decades, especially in the higher frequencies, it is expected that the SRTs in noise for both the WIN and the QuickSIN will increase with the advancing age decade due to the progressively increasing hearing loss. This is confirmed in the middle section of the top panel of Figure 3 (both *F*_(3,133)_ > 14.5, *p* < 0.001). The η^2^ effect sizes were also large for the effect of age group on the WIN SRT (η^2^ = 0.35) and QuickSIN SRT (η^2^ = 0.25). *Post hoc* Bonferroni-adjusted *t*-tests revealed that significant differences were never observed between the mean SRTs in noise for the 50- and 60-year-olds but each of these two age groups had significantly lower SRTs than the 70- and 80-year-olds. In addition, for the WIN, the SRT in noise for the 70-year-olds was significantly better than that of the 80-year-olds.

Regarding the auditory measures of temporal processing, there are no significant effects of age group on gap-detection threshold at either frequency (right section of the top panel). Among the four temporal-order identification measures (bottom panel), only the monaural two-item temporal-order identification task showed a significant effect of age group (*F*_(3,132)_ = 3.8, *p* < 0.0125). For the significant effect of age group on monaural two-item temporal-order identification, the η^2^ value was 0.08 which represents a medium effect size (Cohen, 1988), and *post hoc* Bonferroni-adjusted *t*-tests found that both the 50- and 60-year-olds had lower SOAs than the 80-year-olds. Although it appears that there is a trend toward an effect of the age group for the monaural 4-item and the dichotic-identification task, confirmed by an uncorrected *p* value < 0.05 and medium effect sizes (η^2^ = 0.08 and 0.06, respectively), these differences were not significant following Bonferroni adjustment of the *p* value to 0.0125. The relatively high variability of the oldest group on both tasks may have diminished the effect of the age group for these two measures. It is interesting that on the monaural 4-item and dichotic-location tasks it was the 60-year-olds who had the best performance rather than the 50-year-olds, although this was not a significant difference in SOA between these two age groups. It is unclear from these data whether this pattern for these two measures reflects better-than-expected performance of the 60-year-olds, worse-than-expected performance of the 50-year-olds, or a combination of both.

The mean performance observed here, both overall and by age group, is consistent with prior reports on the hearing threshold (Cruickshanks et al., 1998) and auditory temporal-processing measures (Humes et al., 2009; Harris et al., 2010; Humes and Dubno, 2010; Humes et al., 2013). Given that 101 of the 137 participants in the current study had participated in these earlier studies 9 years previously, the agreement between the present and prior findings on the measures of temporal processing is expected. Regarding the WIN and QuickSIN SRTs in noise, neither having been obtained in our prior studies, the study of Wilson et al. (2007) seemed to be the most appropriate comparison. Wilson et al. (2007) obtained both SRT measures from a group of 72 older adults with hearing loss and from a group of 24 young adults with normal hearing. The older adults in that study were similar in age to the present sample, although the hearing loss was considerably greater and the presentation level about 10 dB lower for the WIN and about 10 dB higher for the QuickSIN. Wilson et al. (2007) presented their SRT data for the entire group of 72 older adults, rather than stratified by age group as in Figure 3. The overall means averaged for the entire group of 137 in this study were 4.0 dB for the WIN and 7.3 dB for the QuickSIN which generally falls between the means for the normal-hearing and hearing-impaired groups for each test in Wilson et al. (2007). More recently, Wilson (2011) reported age-decade WIN thresholds for a large clinical sample with older adults having greater high-frequency hearing loss than those in the present study. Although the average WIN SRT was consistently 4–5 dB higher than observed here (Figure 3), the progression with advancing age was similar. In the present study, the WIN SRT increases in these cross-sectional data at about 2 dB per decade.

**Figure 4:**
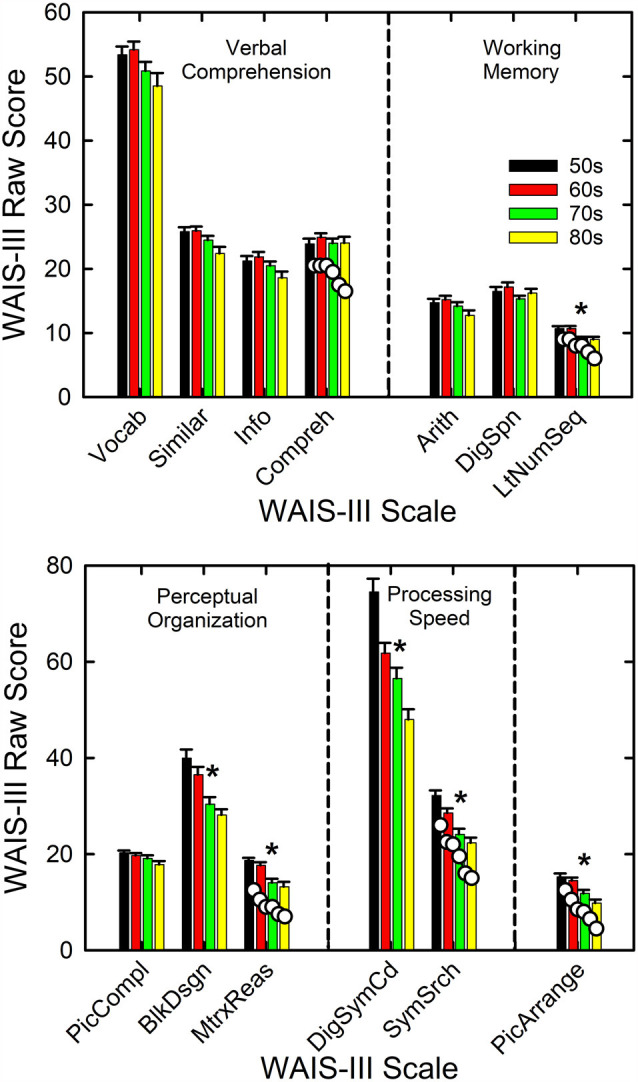
Means and standard errors on the WAIS-III shown by age decade. Measures are partitioned into groups (vertical dashed lines) according to the type of cognitive process. The asterisks mark those measures for which a significant effect of age decade was observed. The white circles shown for the right-most measure in each panel provide example comparisons to normative values from Ardila (2007) for those scales. WAIS-III scales are: Vocab = vocabulary; Similar = similarities; Info = information; Compreh = comprehension; Arith = arithmetic; DigSpn = digit span; LtNumSeq = letter-number sequence; PicCompl = picture completion; BlkDsgn = block design; MtrxReas = matrix reasoning; DigSymCd = digit-symbol coding; SymSrch = symbol search; and PicArrange = picture arrangement.

The primary cognitive measure used in this study was the WAIS-III. Figure 4 shows the means and standard errors for each age decade. The top panel shows the results for the seven scales considered to be verbal in nature, separated into the two domains tapped by those scales (Verbal Comprehension and Working Memory), whereas the bottom panel depicts the results for the performance-based measures, again separated into the two domains tapped (Perceptual Organization and Processing Speed). The Picture Arrangement scale, shown at the far right in the bottom panel, is a performance-based measure like the others in the bottom panel, but it is not included in either of the index scores computed from the other performance-based measures. The asterisks again mark significant effects of age group on the raw scores for each scale in Figure 4 from the GLM analyses (all *F*_(3,133)_ > 6.2) with Bonferroni adjustment of the *p* values (*p* < 0.05/13 or 0.0038). Further, for all six significant effects of age group shown in Figure 4, the η^2^ effect sizes ranged from 0.12 to 0.31, all considered to be large effect sizes (Cohen, 1988). The pattern of age-group effects across the various WAIS-III scales is entirely consistent with expectations (e.g., Salthouse, 2010) and prior findings from a similar cohort (Humes et al., 2013). Specifically, verbal comprehension measures tend not to decline substantially with advancing age in adulthood whereas many process-based measures do decline. Consistent with this expectation, five of the six performance or process-based measures in the bottom panel of Figure 4 show significant effects of age group, as does one of the three measures of working memory (top right). No significant age-group effects were observed for the four measures of Verbal Comprehension in the top left of Figure 4. For the six cognitive measures showing significant effects of age decade in Figure 4, the pattern of age-decade differences, based on *post hoc* Bonferroni-adjusted *t*-tests, was the same for five of the six measures, all but digit- symbol coding. The pattern observed was that the performance of the 50- and 60-year-olds was significantly better than that of the 70- and 80-year-olds with no other significant differences observed. For digit-symbol coding, the 50-year-olds had significantly better performance than all other age decades with the 60-year-olds also outperforming the 80-year-olds significantly.

To illustrate the representativeness of the present data, the mean WAIS-III raw scores from the standardization sample (Ardila, 2007) are shown by the white circles for the far-right cognitive measure in each panel. The age groups for the comparison WAIS-III data are for age groups of 55–64, 65–69, 70–74, 75–79, 80–84, and 85–89 years, with about 190 individuals in each age group. Thus, the comparison data span much of the same range as the age groups in the present analyses but with different groupings of ages. Although the WAIS-III scores from the participants in this study tend to be slightly higher than the group norms from Ardila (2007), likely reflecting a somewhat higher average education level in the present sample, the patterns across age groups are very similar for both datasets.

The other cognitive measure completed in this study was the AQT. As noted previously, the AQT is a brief measure of verbal processing speed. Three primary measures emerge from this test: the time required to complete the color naming only, the shape naming only, or the combined color-shape naming. It has also been suggested that the number of naming errors for the color-shape naming task may be informative. When these four processing-speed measures were analyzed, two of the four, shape-naming time and color-shape-naming time, showed significant effects of age decade with both *F*_(3,133)_ > 6.7, *p* < 0.0125 (or 0.05/4). The η^2^ effect sizes were both > 0.13, moreover, which corresponds to large effect sizes (Cohen, 1988). *Post hoc* Bonferroni-adjusted *t*-tests comparing performance across age decades found that for both the color-shape- and shape-naming times, the 50-year-olds had significantly faster processing speeds than both the 70- and 80-year-olds. For the shape-naming time, the 60-year-olds were also significantly faster than the 80-year-olds. Again, effects of age group on a processing-based measure like the AQT are expected (Salthouse, 2010) and are also consistent with the WAIS-III data shown previously in the bottom panel of Figure 4. When the AQT processing times were averaged across the entire group of 137 adults in this study, the mean times of 23.8 s, 30.4 s, and 56.8 s for the color, shape and color-shape conditions compare favorably to the values of 23.0 s, 29.7 s, and 55.4 s, respectively, reported for a separate group of 98 older adults (Humes et al., 2013).

Finally, for the four visual measures, the TRT and three flicker-fusion contrast thresholds, effects of age group were only observed for the TRT (*F*_(3,131)_ = 16.4, *p* < 0.001). None of the flicker-fusion contrast thresholds showed significant effects of age decade (all *F*_(3,133)_ < 3.3, *p* > 0.0167 or 0.05/3). A very large η^2^ effect size of 0.27 was observed for the TRT. *Post hoc* Bonferroni-adjusted *t*-tests revealed that the performance of the 50- and 60-year-olds, with means of 58 and 58.2% unmasked text at TRT, was significantly better than that of the 70- and 80-year-olds, with means of 61.8 and 62.6% unmasked text at TRT. The overall TRT value averaged across age decades of 60% unmasked text is consistent with that observed in older adults previously (Zekveld et al., 2007; Humes et al., 2013). For the visual flicker-fusion contrast thresholds, the 137 adults in this study had mean contrast ratios of 0.024, 0.01, and 0.006 at 2, 4, and 8 Hz modulation, respectively. The latter two values are in line with prior findings from a large group of older adults, but the contrast threshold at 2 Hz is somewhat larger than observed previously (Humes et al., 2009).

All told, across the various sensory and cognitive measures obtained, the results from the 137 adults in this study compare favorably to those obtained from groups of similar age and with similar amounts of hearing loss. The patterns observed across age decade for auditory, cognitive, and visual measures were also compatible with expectations and available literature. The WIN and QuickSIN SRT values are also in line with prior observations from comparable participants. Thus, the performance of the present sample of 137 older adults can be considered representative of older adults with similar demographics.

### Individual Differences in SRT in Noise

Prior to performing the linear-regression analyses for the WIN and QuickSIN SRTs, a series of principal-components factor analyses (Gorsuch, 1983) were completed to reduce the set of independent variables by eliminating redundancy within each set. For the cognitive measures, the 13 WAIS-III scale scores, shown previously in Figure 4, and the four AQT measures were analyzed using factor analysis with an extraction criterion of eigenvalue > 1.0. A good fit was obtained with four factors accounting for 67.9% of the variance. All communalities exceeded 0.48 and most were above 0.6. The Kaiser-Mayer-Olkin (KMO) measure of sampling adequacy was 0.87 also supporting a good fit to the data. The four components were rotated orthogonally using the Varimax criterion which resulted in a clear interpretation of each factor. For example, the digit-symbol coding score and the symbol-search score from the WAIS-III loaded heavily and positively on the first component as did the three time-based measures from the AQT (loading negatively in this case). This first component represents processing speed (PS) and is referred to here as W3aqtPS. In a similar fashion, the other three components were identified as W3VC, W3WM, and W3PO, representing verbal comprehension (VC), working memory (WM), and perceptual organization (PO) scales of the WAIS-III, respectively, corresponding to the partitioning of scale scores into the functional categories shown in Figure 4. The AQT error score for the color-shape identification task loaded, again negatively, with the three WAIS-III tests comprising the PO factor, which loaded positively on this factor.

Principal-components factor analysis was next conducted for the nine psychophysical measures of auditory function in this study. A good fit was obtained with three orthogonal factors explaining 64.4% of the variance, all communalities exceeding 0.38, and the KMO sampling adequacy = 0.58. The three factors were identified easily as pure-tone threshold (AudPT), gap-detection and temporal-order identification (AudGDTO), and dichotic temporal-order processing (AudTOdich). The distinction between the latter two factors is that the SOA for the temporal-order dichotic-location task (identify the ear) only loaded on this latter factor whereas the other dichotic temporal-order task (identify the vowel) loaded moderately on both the AudGDTO and the AudTOdich factors.

For the visual flicker-fusion contrast thresholds, the thresholds for all three flicker rates were reduced *via* principal-components factor analysis to a single factor accounting for 64.4% of the variance. The KMO sampling adequacy measure was 0.57 and all communalities were greater than 0.45. This factor is referred to as VisFF here.

Several other predictors or independent variables to be included in the linear regression analyses were first converted to z-scores. This transformation resulted in means of 0 and standard deviations of 1 for these measures, the same means and standard deviations for each of the factor scores noted above. The measures undergoing *z*-transformation and the labels used here for each were age (zAge), PTA4 (zPTA4), TRT (zTRT), SII for the WIN (zSIIwin), and SII for the QuickSIN (zSIIqsin). The two dependent measures, QuickSIN and WIN SRT, were also z-transformed (zWIN and zQuickSIN, respectively). Except for age and the TRT, all measures are for the right ear, as was the case for all the psychophysical measures in this study except the dichotic measures which clearly involved both ears.

The correlations between the various measures of “audibility” were examined next and, not surprisingly, the zPTA4 and the AudPT factor score were strongly correlated (*r* = 0.92, *p* < 0.01). Given the more widespread usage of PTA4 in the literature, zPTA4 was used in the subsequent regression analyses. As expected, the correlations of PTA4 with the SII were moderate to strong and significant (*p* < 0.001) with *r* = −0.61 and −0.82 for the QuickSIN and WIN, respectively. In addition, given the weaker correlations of PTA4 with the SII compared to the correlation between the clinical and laboratory measures of hearing loss, correlation magnitudes of 0.6–0.8 vs. 0.9, both zPTA4 and the corresponding SII measure will be included in subsequent regression analyses.

To capture the audibility deficit for each SRT in noise measure, the SII was first calculated at the normal-hearing SNR value of +4 dB for both the WIN and QuickSIN tests. The SII value for normal-hearing young adults at +4 dB SNR corresponds to 0.549 for the QuickSIN with SII values of 0.628 and 0.589 for the 93- and 103-dB SPL presentation levels of the WIN, respectively. Next, the 0 dB-HL hearing thresholds of young normal-hearing adults were replaced by those of the older adult and a reduced SII was most often observed. This is the SII used in the regression analyses and represents the reduction in audibility from hearing loss. When the SII had been reduced by the hearing loss, the SNR was then increased in steps of 0.1 dB until the SII for the older adult matched that of the young normal-hearing reference group (e.g., SII = 0.549 for the QuickSIN). In other words, the SNR needed to compensate for the audibility loss in the higher frequencies was established for each individual and for each speech-in-noise measure. This is referred to here as the SNRsii or the SNR needed to equate SII values to that measured in young normal-hearing listeners at +4 dB. It should also be noted that for the WIN, which varies the speech level to obtain the SNR for a fixed noise level, the level desensitization component of the SII calculations also influenced the SNR needed to match the reference SII calculated for young normal-hearing adults.

**Figure 5:**
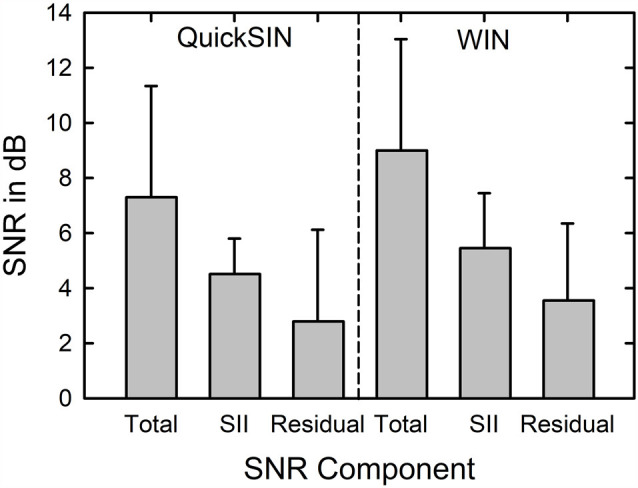
Means and standard deviations (error bars) for the SNRtotal, SNRsii, and SNRresidual components for the 137 older adults in this study for the QuickSIN (left) and WIN (right). SNRtotal is the measured SNR at SRT, SNRsii is the SNR needed to achieve the normal-hearing SII for each listener, and SNRresidual = SNRtotal − SNRsii.

When the SNRsii was compared to the measured SNR for each test, most often the measured or total SNR exceeded the SNRsii value. When this was the case, this implies that the measured SNRtotal is not solely attributable to the loss of audibility. The extra SNR improvement needed is referred to here as SNRresidual. In this way, the measured SNR for each test, SNRtotal, could be partitioned into two components, SNRsii and SNRresidual. That is, SNRtotal = SNRsii + SNRresidual. Means and standard deviations for each of the SNR components, total, sii, and residual, are shown in Figure 5 for the QuickSIN and WIN. Although the SNRsii represents a substantial portion of the measured SNRtotal, the mean SNRresidual is 3–4 dB for both speech-in-noise measures. Each residual is significantly greater than 0 dB [both *t*_(136)_ > 9.8, *p* < 0.001]. The correlations of each SNRresidual with the measured SNRtotal for each test were also strong and significant (*r* = 0.89 for the WIN and *r* = 0.96 for the QuickSIN, *p* < 0.001 for both). The correlations between SNRsii and SNRresidual, although significant (*p* < 0.001), were moderate for the WIN (*r* = 0.40) and QuickSIN (*r* = 0.41) reflecting the relative independence of these two components of the SNRtotal. Also, as discussed in more detail below, there is no direct one-to-one correspondence between the decomposition of SNRtotal into SNRsii and SNRresidual and the decomposition of SRT-in-noise into attenuation and distortion terms in Plomp’s SRT model. As noted below, several individuals with apparent “SNR Loss” or “distortion” terms do not have significant SNRresidual components.

Linear regression analyses were performed next for each of the z-transformed QuickSIN and WIN SNR components. Table 2 shows the results of the stepwise regression analysis for each SNR component of the QuickSIN. The criterion *p*-values for progression through the steps were *F* values with *p* < 0.05 for inclusion and *p* > 0.1 for exclusion. The stepwise solution was also compared to the solution for entry of all predictor variables in the equation and the significant independent variables were the same with either approach giving more confidence in the stepwise solution (Chowdhury and Turin, 2020). As shown in the top portion of Table 2, four variables were identified as significant predictors of the measured QuickSIN SNRtotal (*F*_(4,132)_ = 20.3, *p* < 0.001) collectively accounting for 59.6% of the variance in QuickSIN SRT. Assuming a test-retest correlation of about *r* = 0.9 for the QuickSIN, comparable to that observed for the WIN (Wilson and McArdle, 2007), the maximum systematic variance that could be accounted for is 81% (*r*^2^). In this light, accounting for 59.6% of the total variance, or 73.6% of the systematic variance is a very good fit. The variance inflation factors (VIFs), moreover, were all less than 2.2 and the Condition Index values less than 2.6, both indicating that collinearity among the independent variables was not an issue. This is also supported by the partial and part (semi-partial) correlations in the top portion of Table 2. Partial correlation is the correlation between an independent variable and a dependent variable after controlling for the influence of other independent variables on *both* the independent and the dependent variable. The part or semi-partial correlation does not control for the influence of the other independent variables on the dependent variable, only on the independent variable.

**Table 2:** The results of the linear regression analyses for the *z*-transformed QuickSIN SNR components at SRT.

Dependent variable	*r*^2^	*r*^2^change	Predictor variables	Beta	*t*	*r*	Partial *r*	Part *r*
zSNRtotal	0.596	0.461	zPTA4	0.340	4.24	0.679	0.346	0.234
		0.103	zSIIqsin	−0.394	−5.64	−0.668	−0.441	−0.312
		0.018	zAge	0.154	2.19	0.538	0.187	0.121
		0.013	W3VC	−0.116	−2.08	−0.159	−0.178	−0.115
zSNRsii	0.984	0.983	zSIIqsin	−1.002	−83.0	−0.992	−0.990	−0.911
		0.001	zAge	−0.025	−2.08	0.391	−0.177	−0.023
zSNRresidual	0.418	0.350	zPTA4	0.436	5.15	0.591	0.409	0.342
		0.031	zAge	0.210	2.49	0.501	0.212	0.165
		0.020	W3VC	−0.142	−2.12	−0.187	−0.182	−0.141
		0.017	W3WM	−0.134	−1.99	−0.216	−0.170	−0.132

*Stepwise regression analyses were performed and all predictor variables shown were significant (*p* < 0.01)*.

The significant predictor variables for zQuickSIN SNRtotal include two auditory-based measures (zSIIqsin, zPTA4), age, and one cognitive measure. It is noteworthy that both hearing loss, zPTA4, and audibility as captured by the SII, zSIIqsin, emerged as significant predictors of measured QuickSIN performance. Further, based on the correlations in Table 2, the contributions of each are strong and roughly equal whereas the other two significant predictors make smaller contributions. Note that the zSIIqsin predictor variable has a negative Beta coefficient. This reflects the needed increase in SNR resulting from hearing loss with more severe hearing loss yielding lower SII values and a need for higher SNRs to compensate for this loss of audibility. The more the SII has been reduced by the presence of the hearing loss, the more the SNR needed to be increased to achieve the targeted (normal) SII. As a result, the correlation between zSIIqsin and zSNRsii is nearly perfect (*r* = −0.99) for the QuickSIN, as expected. As a result, either term could be used interchangeably in the regression analyses with the SII used here as this is the direct measure of inaudibility upon which the SNRsii is based.

For hearing loss and age, the higher the value of either, the greater the SNRtotal that was measured on the QuickSIN. Those with greater hearing loss, *even after factoring in the loss of audibility *via* the SII*, required higher SNRs, as did those who were older. The negative Beta coefficient for the cognitive measure, W3VC, indicates that the higher the cognitive function, the lower (better) the SNR for the QuickSIN. The association of SNRtotal on the QuickSIN with verbal comprehension (VC) likely reflects the use of meaningful sentences in the QuickSIN.

The remainder of the entries in Table 2 show the stepwise regression results for the two SNR components, SNRsii and SNRresidual. Not surprisingly, given the trade-off between SII due to hearing loss and the amount the SNR is then increased to reach the SII target, SIIqsin and SNRsii should be strongly correlated. A total of 98.4% of the variance in zSNRsii was explained with almost all of it explained by zSII. Perhaps more interesting are the regression results for SNRresidual in the bottom portion of Table 2. Several things are noteworthy about these analyses. First, note that 41.8% of the total variance is all that could be explained given the auditory, visual, and cognitive measures available. Although this is still significant and impressive, it is much lower than the amount of variance explained for either the SNRtotal or the SNRsii. Second, note that, although zPTA4 is a strong predictor, accounting for most of the explained variance in SNRresidual, zSIIqsin is *not* a significant predictor. This reinforces that the SNRresidual component is not simply another manifestation of inaudibility. Rather, the severity of hearing loss, PTA4, appears to be a marker for poor processing of the stimuli such that the more severe the hearing loss, the more the SNR must be increased *beyond that attributable to inaudibility alone*.

A review of the results of all three regression analyses in Table 2 for the QuickSIN indicates that the two primary predictors of the measured SNRtotal are the SII and PTA4, each making separate and distinct contributions. Further, age and cognitive function also emerge as statistically significant predictors for SNRtotal and these predictors make the strongest contributions to the SNRresidual component of the measured SNRtotal. The contributions made by age and cognition, however, are quite small, typically accounting for less than 3% of the total variance in SNRtotal or SNRresidual. Finally, note that the regression analysis for SNRtotal basically reveals a composite of the factors predicting each of the SNR components, SNRsii and SNRresidual.

Next, an identical set of linear regression analyses were conducted with zSNRtotal for the WIN as the dependent variable. Table 3 shows the results for each of the three SNR components for the WIN, SNRtotal (top), SNRsii (middle), and SNRresidual (bottom) with 76.8%, 96.7%, and 46.3% of the total variance explained for each SNR, respectively. The pattern of results in Table 3 is remarkably similar to that for the QuickSIN in Table 2. This is true for the percentage of total variance explained in each of the three regression analyses as well as in the nature of the specific significant predictors that emerged in each analysis. Although hearing loss (PTA4), audibility (SIIwin), age, and cognition are again identified as the key predictors for various SNR components, two main differences emerged for these analyses of the WIN compared to those described previously in Table 2 for the QuickSIN. First, the specific cognitive predictor was verbal comprehension (W3VC) for the QuickSIN (Table 2) but is working memory (W3WM) for the WIN (Table 3). Second, an additional predictor variable, visual flicker fusion (VisFF), emerged in the analyses of the WIN SNR components. This variable, however, only accounted for 2.2–3.6% of the total variance in SNRtotal and SNRresidual, respectively.

**Table 3:** The results of the linear-regression analyses for the z-transformed WIN SNR components at SRT.

Dependent variable	*r*^2^	*r*^2^ Change	Predictor variables	Beta	*t*	*r*	Partial *r*	Part *r*
zSNRtotal	0.768	0.688	zSIIwin	−0.453	−5.9	−0.829	−0.459	−0.248
		0.033	zPTA4	0.285	3.7	0.782	0.311	0.158
		0.012	W3WM	−0.134	−3.1	−0.271	−0.261	−0.130
		0.014	zAge	0.205	3.6	0.617	0.302	0.152
		0.022	VisFF	−0.155	−3.5	−0.044	−0.292	−0.147
zSNRsii	0.967	0.951	zSIIwin	−1.123	−39.0	−0.975	−0.959	−0.618
		0.014	zPTA4	−0.194	−6.9	0.728	−0.515	−0.110
		0.002	W3WM	0.042	2.5	0.306	0.215	0.040
zSNRresidual	0.463	0.370	zPTA4	0.440	5.40	0.608	0.425	0.344
		0.026	zAge	0.272	3.28	0.498	0.274	0.209
		0.036	VisFF	−0.208	−3.14	−0.072	−0.263	−0.200
		0.031	W3WM	−0.179	−2.77	−0.253	−0.234	−0.177

*Stepwise regression analyses were performed and all predictor variables shown were significant (*p* < 0.05)*.

For the regression analyses of zSNRtotal for the WIN (top portion of Table 3), the significant regression solution (*F*_(5,131)_ = 20.9, *p* < 0.001) accounted for 76.8% of the total variance. Again, assuming a test-retest correlation of *r* = 0.9 for the WIN (Wilson and McArdle, 2007) or 81% of the total variance being systematic non-error variance, this solution accounts for 94.8% of the systematic variance representing an excellent fit to the data. There were no indications of collinearity among the independent variables with all VIF values <3.4 and Condition Index values <3.8. As was true for the QuickSIN (Table 2), the predominant predictor variables for SNRtotal, SNRsii, and SNRresidual are the SII and PTA4, with each of the other variables typically explaining less than 4% of the total variance. In general, the lower the SIIwin, working memory score, or visual flicker threshold and the greater the PTA4 or age, the higher the SNR for the WIN. Of these predictors, the only one that is unexpected is the inverse association between SNRtotal and visual flicker fusion (VisFF). The higher (worse) the visual contrast needed for flicker fusion the lower (better) the SNR.

## General Discussion

The first portion of the data analyses sought both to establish that the present data are consistent with those in the literature and to develop some normative values for each age decade from the 50s through the 80s. The latter goal was especially important for the auditory temporal-processing measures and the two SRT measures (QuickSIN and WIN) as such age-specific norms had not been published previously except for the WIN in a large clinical dataset (Wilson, 2011). Age-specific norms for pure-tone audiometry and the WAIS-III, on the other hand, are readily available and have been replicated many times. Where comparisons were available, these initial analyses demonstrated that the data gathered from the current sample of 137 older adults were consistent with the literature and with the expectations generated from that literature regarding age effects.

This study also found that, when relatively high speech and noise levels were used with older adults, individual differences in the SNR for 50%-correct speech-recognition performance, or SRT, were largely explained by four factors. These factors included the SII, a measure of average hearing loss (PTA4), age, and cognitive processing (verbal comprehension for the QuickSIN and working memory for the WIN). The variance accounted for by the regression solutions for the QuickSIN and WIN, moreover, appeared to capture most (74%–95%) of the systematic or non-error variance in these measures.

**Figure 6:**
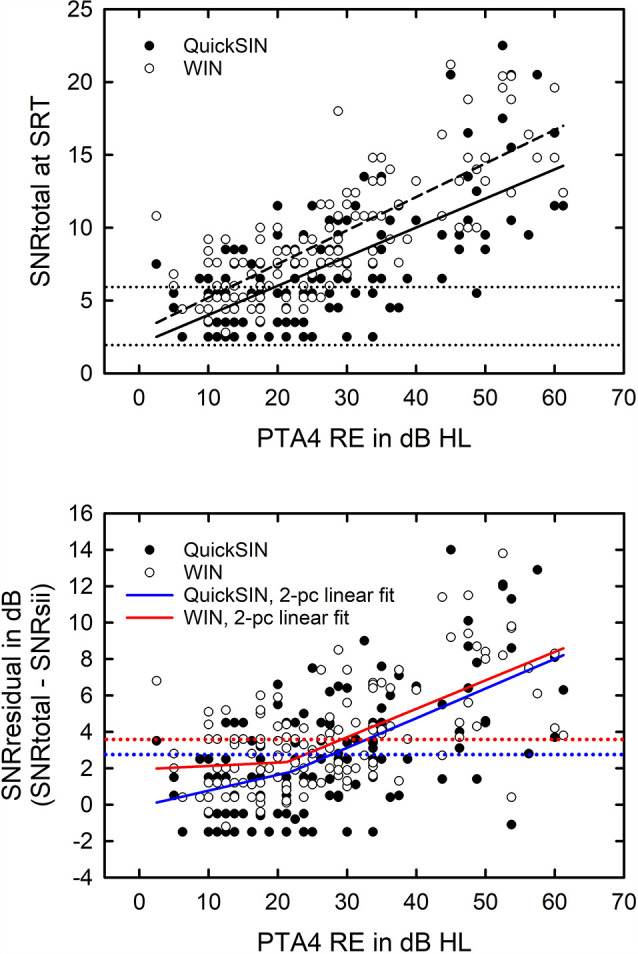
The top panel shows the measured signal-to-noise ratio (SNR), SNRtotal, for the QuickSIN (filled circles), and the WIN (unfilled circles) for the 137 older adults in this study plotted as a function of the pure-tone-average for 500, 1,000, 2,000, and 4,000 Hz in the right ear (PTA4 RE). The sloping solid and dashed lines are the best-fitting linear-regression solutions for the QuickSIN (solid; *r* = 0.68) and WIN (dashed; *r* = 0.78). Assuming a mean SNR at SRT for young normal-hearing adults of +4 dB and a standard deviation (SD) of 2 dB for both tests, the dotted horizontal lines represent ± 1 SD boundaries for these norms. The bottom panel plots the difference, SNRresidual, between the measured SNRs at SRT, SRTtotal, in the top panel and the SNR at SRT predicted by the SII from the hearing loss, SNRsii, plotted as a function of PTA4 RE. The best-fitting two-piece linear fits for SNR residual are plotted for the QuickSIN (blue solid; *r* = 0.59) and WIN (red solid; *r* = 0.61). The dotted horizontal lines represent the upper bound of the 95% confidence interval from test-retest measures for each test, 2.7 dB for the QuickSIN (blue dotted line), and 3.6 dB for the WIN (red dotted line).

At first glance, it may be somewhat surprising to find that the two best predictors of individual differences in SRT in noise were the SII and PTA4 as both can be considered measures of the audibility of the speech and noise stimuli. As noted in the preceding section and in the introduction, however, although moderately correlated, these two measures are *not* equivalent and each appears to capture variance in SRT performance not captured by the other. This was also supported through the regression analyses of the SRTtotal, SRTsii, and SRTresidual components for each speech-in-noise measure (Tables s 2, 3). Figure 6 provides a more detailed examination of the co-emergence of both SII and average hearing loss as major predictors of speech-in-noise performance. In the top panel of Figure 6, the SNRtotal at SRT for the QuickSIN (filled circles) and WIN (unfilled circles) are plotted as a function of the PTA4 in the right ear. The correlations between the SRT and PTA4 are *r* = 0.68 and *r* = 0.78 for the QuickSIN and WIN SNRtotal values, respectively, consistent with correlation magnitudes observed for both measures previously (Hanna and Robinson, 1985; Killion et al., 2004; Wilson et al., 2007; Wilson, 2011; Williams-Sanchez et al., 2014). Best-fitting linear-regression fits for each test in the top panel of Figure 6, a dashed line for the WIN and a solid for the QuickSIN, show the clear dependence of both SRT measures on PTA4 as has been observed for both tests previously using various measures of pure-tone average (Killion et al., 2004; Wilson et al., 2007). Based on the data in the literature for both tests, the mean (M) and standard deviation (SD) for the SNR at SRT in young normal-hearing listeners are well approximated by values of 4.0 and 2.0 dB, respectively (Killion et al., 2004; McArdle et al., 2005; Wilson et al., 2007). The horizontal dotted lines in the top panel of Figure 6 are the boundaries resulting from M ± 1 SD and the upper boundary at an SNR value of 6 dB well approximates the upper limit of “normal hearing” suggested for both tests (Etymotic Research, 2001; Wilson and Burks, 2005; Wilson, 2011). Of the 137 subjects, using this definition of normal performance, 69 (50.4%) have SNRtotal values at SRT for *both* the QuickSIN and the WIN that exceed the 6-dB upper limit. Thus, from consideration of the SRT in noise alone, half of the participants would be considered to have significant “distortion” or an “SNR loss” (Plomp, 1978; Etymotic Research, 2001; Killion et al., 2004); that is, half required a significantly greater-than-normal SNR to reach 50% correct recognition in noise.

This is illustrated further in the bottom panel of Figure 6. Here, the SII was used to predict the SNR at SRT for both the QuickSIN and the WIN based on the hearing loss alone (SNRsii) with the difference between the measured (SNRtotal) and audibility-based SNR (SNRsii), the SNRresidual, plotted as a function of PTA4 in the bottom panel of Figure 6. A 0-dB residual would be interpreted as the SII completely explaining the measured SNRtotal for each test. Each test, however, has some inherent measurement error. For the SRTs measured with one list of the QuickSIN, as in this study, the 95% confidence interval for test-retest is 2.7 dB (Killion et al., 2004) and this is shown in the bottom panel of Figure 6 as the blue dotted line. The corresponding value for the WIN is 3.6 dB (Wilson and Burks, 2005) and this appears as the red dotted line in the bottom panel of Figure 6. The blue and red solid lines show the best-fitting two-piece linear fits for the QuickSIN and WIN, respectively. The two-piece linear functions accounted for 36%–39% of the variance which was slightly better than the linear fits (34%–37% of the variance explained). Note that the points at which the red solid and dotted lines, as well as the blue solid and dotted lines, intersect is at PTA4 values of 28–30 dB HL. In addition, the inflection point of the two-piece linear best fits occurs at a PTA4 value of about 22 dB HL for both the QuickSIN and WIN. Above the point of intersection or the point of inflection for each test, basically for PTA4 ≥ 25–30 dB HL, the SRTresidual increases steadily with increasing PTA4. Again, it should be kept in mind that this is *after* the inaudibility of the speech and noise stimuli has been taken into consideration *via* the SII and that stimulus levels used here were 10-dB greater than those recommended for clinical use.

**Figure 7:**
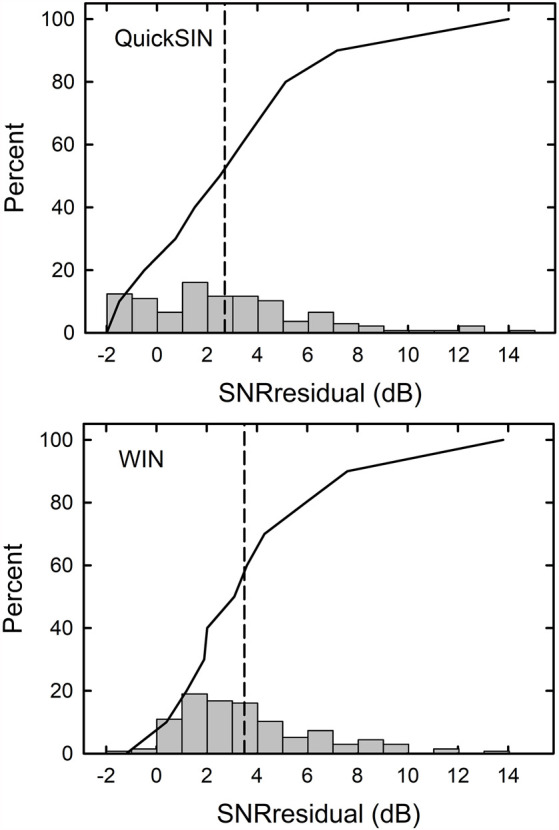
Histograms (gray vertical bars) and cumulative distributions of the SNRresidual values for the QuickSIN (top) and WIN (bottom) for the 137 older adults in this study.

As indicated in the bottom panel of Figure 6, PTA4 is clearly related to the magnitude of the measured SNR elevation, SNR residual, for those with PTA4 exceeding 30 dB HL. The residual errors from the SII prediction are not random but depend systematically on PTA4 for both the WIN and the QuickSIN with the best-fitting two-piece linear fits explaining 36–39% of the variance. Figure 7 shows the distribution of the SNRresidual values for the QuickSIN (top) and WIN (bottom). The solid line in each panel represents the cumulative distribution for each SNRresidual and the vertical dashed lines are the 95% confidence intervals for test-retest noted previously for each test. For the QuickSIN, 56.2% of the residual values are within the 95% confidence interval of a 0-dB residual and, for the WIN, 54.7% of the values fall within the 95% test-retest confidence interval. Thus, for a little over half of the older adults in this study, the SNRtotal was within the test-retest variability of the SNRsii and could be explained entirely by the audibility loss. For the remaining 45%, however, this was not the case and significant residual SNRs were observed on one of the speech-in-noise measures.

Although about 45% had SNRresidual values exceeding the test-retest confidence interval indicating that more than inaudibility may be contributing to their SNRtotal, it was not the same individuals who demonstrated a significant SNRresidual on both the WIN and QuickSIN. In fact, there were 41 of the 137 older adults whose SNRresidual values for *both* the WIN and the QuickSIN significantly exceeded 0 dB. Such co-occurrence of a higher-than-expected SNR for both speech-in-noise measures provides greater confidence in the conclusion that the higher SNR provided a valid indication of those older adults with speech-in-noise difficulties.

As explained previously, the SII is essentially a weighted integration of the SNR over the frequency range of about 250–8,000 Hz with the effective range of the SNR set to ±15 dB relative to the long–term speech spectrum. In principle, if portions of the speech spectrum are rendered inaudible through low-pass filtering or, in this case, severe high-frequency hearing loss, this can be compensated for by increasing the SNR over the frequency region that remains audible (Humes and Dubno, 2010). The inability of the SII predictions to fully account for the measured SRT in noise for both speech-in-noise measures for 41 of the 137 (29.9%) older adults could reveal a limitation of the SNR-bandwidth trade-off inherent to the SII. Alternately, the greater severity of the high-frequency hearing loss may serve as a marker for the severity of cochlear pathology that impacts more than the hearing threshold (Humes, 2007). It has been suggested previously, for example, that thresholds exceeding 60 dB HL may imply underlying cochlear pathology beyond the loss of outer hair cells, including loss of inner hair cells or dysfunction of nerve fibers (e.g., Moore, 2004; Aazh and Moore, 2007). Perhaps such factors underlie the systematic dependence of the SNRresidual on PTA4 observed for both tests in the bottom panel of Figure 6. Of course, it is also possible that the 41 older adults with poorer than predicted speech-in-noise performance on both tests in this study had other types of deficits, such as deficits in temporal processing or cognitive function, that necessitated substantially higher SNRs for both the WIN and the QuickSIN.

The linear-regression analyses summarized previously in Tables s 2, 3 revealed that the strongest contributors to individual differences in SRTresidual, beyond PTA4, were cognitive in nature; specifically, working memory (WIN and QuickSIN) and verbal comprehension (QuickSIN only). The measures of auditory temporal processing examined here did not contribute significantly to the regression solutions for either speech-in-noise measure. Of course, there could be some other suprathreshold auditory processing deficit, not measured here and correlated with the severity of underlying cochlear pathology (PTA4), underlying such difficulties.

A key finding in this study concerns the important role played by consideration of the influence of the SII on the measured SRTtotal for each test. For example, of the 77 older adults with “SNR Loss”, a SNRtotal >6 dB, on the QuickSIN, 17 or 22%, did not have a significant SNRresidual component. In other words, for 22% of those with an “SNR Loss” on the QuickSIN, the loss could be attributed to inaudibility. For the WIN, 95 of the older adults would be identified as having “SNR Loss” but 33 of these individuals (35%) did not have significant SNRresidual components suggesting that the measured “SNR Loss” was attributable to inaudibility for these 33 older adults. Finally, as noted, the presence of “SNR Loss” on both tests yields a more robust determination of such loss. Of the 69 older adults with such loss on *both* speech-in-noise tests, 28 (41%) did not have significant SNRresiduals on both SRT measures whereas the remaining 41 older adults had significant SNRresiduals. If one accepts the more robust estimate of such speech-in-noise difficulty based on poor performance on *both* measures of speech-in-noise, then 41 of the 137 older adults in this study, 29.9%, would be considered to have significant “distortion” or “SNR Loss”, down from the 50.4% estimated prevalence of such difficulties without taking SII-based inaudibility into consideration. Again, it should be kept in mind that the presentation levels used here were increased to be 10-dB higher than recommended for clinical use in an effort to minimize the inaudibility of the stimuli. It is likely that the proportion of those with significantly elevated SRTs in noise would have been higher had lower presentation levels been used. It is also possible that a higher percentage of those elevated SNRs may have been attributable to SII-based inaudibility of the stimuli at those lower stimulus levels.

Hearing aids represent the most common intervention for older adults with hearing loss and are designed to improve speech communication in noise. For the most part, hearing aids compensate for the inaudibility of the speech stimulus and can improve speech-in-noise performance considerably as a result (Humes and Dubno, 2010). This improvement will be more challenging to attain, however, for those with significant SNRresidual components. Here, the devices will need to also improve the SNR acoustically through directional microphones or noise-reduction processing. Auditory training to make better use of the existing SNR may also prove beneficial (Ferguson et al., 2014; Henshaw et al., 2015). To the extent that deficits in cognitive function underlie the SNRresidual component of speech-in-noise performance in older adults, computer-based auditory training might prove useful as improvements in cognitive functions pertinent to speech-in-noise performance have been demonstrated for such training (Ferguson et al., 2014; Ferguson and Henshaw, 2015a, b).

Future studies of speech-in-noise performance using SRT measures such as the WIN and QuickSIN should make use of measures like the SII to control for audibility then, after doing so, may proceed to the identification of factors beyond audibility that influence performance. When doing so here, the linear-regression analyses presented in Tables s 2, 3 indicated that the individual differences in SNRresidual were captured by a combination of the severity of hearing loss and cognitive processing.

## Data Availability Statement

The raw data supporting the conclusions of this article will be made available by the author, without undue reservation.

## Ethics Statement

The studies involving human participants were reviewed and approved by Indiana University-Bloomington IRB. The patients/participants provided their written informed consent to participate in this study.

## Author Contributions

LH oversaw the research study, performed data analyses, wrote the manuscript, and approved it for publication.

## Conflict of Interest

The author declares that the research was conducted in the absence of any commercial or financial relationships that could be construed as a potential conflict of interest.
